# Carbon emission reduction and profit distribution mechanism of construction supply chain with fairness concern and cap-and-trade

**DOI:** 10.1371/journal.pone.0224153

**Published:** 2019-10-29

**Authors:** Wen Jiang, Li Yuan, Lanjun Wu, Shiyue Guo

**Affiliations:** 1 College of Architecture and Urban-Rural Planning, Sichuan Agricultural University, Chengdu, P.R. China; 2 Southwest Oil & Gas field Company, Petro China, Chengdu, P.R. China; Sunway University, MALAYSIA

## Abstract

Fairness concern behavior is extremely common in social life, and many scholars are beginning to pay attention to this behavior. In this study, we investigate a two-echelon construction supply chain that consists of a general contractor and a subcontractor under cap-and-trade policy. We study the carbon emission reduction decisions and profit distribution mechanism in the construction supply chain with fairness concern and cap-and-trade. We use the Nash bargaining model to describe the fairness concerns of the construction supply chain members and use the co-opetition model to portray the profit distribution. We show that the fairness concern can impose an adverse influence on firms’ profits and decrease the magnitude of their carbon emission reductions. The subcontractor’s fairness concern causes greater losses to the construction supply chain’s profit. We further demonstrate the impact of fairness concern on the optimal decisions of the general contractor and the subcontractor through numerical analysis.

## Introduction

In recent years, global warming has become increasingly serious and environmental problems such as rising sea levels and frequent extreme weather caused by climate change have become increasingly prominent. Many studies have shown that the large amount of carbon dioxide and other greenhouse gas emissions are the main causes of global warming[1,2]. In 2018, the total amount of global carbon emissions reached 31.1 billion tons[3], and China’s total amount of carbon emissions reached 10 billion tons[4]. It is acknowledged that construction activity greatly impacts on the environment. However, construction industry enterprises face many problems, such as inefficiency, waste and pollution, due to the lack of scientific and effective management[5,6]. Supply chain management provides a useful method for optimizing the construction process[7]. From the perspective of construction supply chain management, studying the operational decision-making of construction supply chain enterprises facing the pressure to protect the environment has become a hot issue[8].

To protect the environment, governments are seeking different solutions. Cap-and-trade policy has become the most effective mechanism for curbing carbon emissions[9,10]. The policy achieves the goals of economic development and carbon emission reduction through both government and market regulation. The main function of cap-and-trade is setting a certain carbon emission cap to a single emission entity for a given period of time. With the promotion of this policy, construction supply chain parties face many challenges. These parties are independent economic entities and determine their own level of carbon emission reduction efforts with the goal of maximizing their own profits. With the implementation of the cap-and-trade policy, firms must determine their own emission reduction efforts and how to distribute benefits between construction supply chain parties under complex decision constraints (capacity, capital and policy). In a construction supply chain, reasonable allocation of carbon emission reduction benefits is a key issue that needs to be addressed. A reasonable profit distribution mechanism is an important way to coordinate the objectives of the supply chain parties and motivate both parties to improve their level of carbon emission reduction efforts. In recent years, many studies (Fehr and Schmidt(1999)[11]; Liu(2018)[12]; Du(2018)[13]; Pu(2019)[14]) have found that many supply chain parties (such as the general contractor and the subcontractor) exhibit fairness concern in practice. They not only care about their own interests, but also about the interests of other parties [15]. The general contractor (GC) and the subcontractor (SC) also compare their status, income and other aspects. If a firm in the supply chain feels that the profit distribution is unfair, they may reduce their efforts and even refuse the program[16,17]. This behavior is defined as fairness concern[18]. On the one hand, existing studies on the decision-making of construction supply chain firms under the cap-and-trade policy are limited; on the other hand, they also lack attention to the fairness concern behavior of the construction supply chain firms. Therefore, this study will address the following questions to fill this gap in the literature:

1. Under cap-and-trade policy, how can construction supply chain firms make carbon emission reduction decisions considering the fairness concern?
2. Under cap-and-trade policy, how can profit be distributed among the construction supply chain firms considering the fairness concern so as to maximize the total profit of the construction supply chain?
3. How does the fairness concern affect the decision of the GC and the SC?

To solve the problems above, this study combines the typical characteristics of low-carbon construction under cap-and-trade policy (carbon emission reduction input can also allow firms to obtain carbon emission reduction income) and introduces construction supply chain management into construction project management. Based on the background above, this study examines carbon emission reduction and profit distribution mechanism in a construction supply chain with fairness concern and cap-and-trade.

The remaining of this article is organized as follow. After a brief Literature review, we present model descriptions and assumptions. In Base model section, we study the profit distribution mechanism and the GC’s and the SC’s carbon emission reduction decisions without fairness concern. In Decision model with fairness concern section, the GC’s and the SC’s carbon emission reduction and the profit distribution ratio are obtained in the condition of considering the SC has fairness concern, the GC has fairness concern and both sides have fairness concern respectively. In Numerical analysis section, we illustrate the impact of fairness concern on firm’s decision-making and profits. Finally, we point out management recommendations and work that requires further research.

## Literature review

Based on the research content of this study, our work is related to following three streams of literature. The first stream investigates operational decisions under cap-and-trade policy; the second stream studies operational decisions considering fairness concern and the last stream explores the incentive mechanism of construction supply chain.

### Operational decisions under cap-and-trade policy

Some scholars have studied the decisions under the cap-and-trade policy from a single perspective (Wang(2017)[19]; Yang(2018)[20]). However, closely related to the research in this study is the research on the operational decisions of supply chain firms based on the perspective of supply chain management. Research shows that the existence of the external carbon trading market will change the structure of the supply chain to some extent[21]. Moreover, studying carbon emissions from a supply chain perspective can not only achieve better carbon emission reduction, but also create new value for supply chain firms[22]. Studies about decision-making in supply chain companies under cap-and-trade usually use the stackelberg model. Du et al. (2013) focused on an emission-dependent supply chain consisting of a manufacture and a supplier, the study investigated their optimal decisions (product quantity and emission permits pricing) in Stackelberg game[23]. Xu et al. (2018) focused on a Make-To-Order supply chain consisting of a manufacturer and a retailer, construed a Stackelberg model with the manufacture as a leader and studied the production and emission abatement decisions of both sides under cap-and trade policy[24]. As object of their study, Xia et al. (2018) took a dyadic supply chain in which a single manufacturer plays a Stackelberg game with a single retailer and incorporated reciprocal preferences and consumers’ low-carbon awareness (CLA)[25]. Wang et al. (2018) studied carbon emissions reduction with cap-and-trade policy and consumers’ low-carbon preference in a dual-channel supply chain. The study examined the pricing strategies and profits of the supply chain members by creating a Stackelberg game model[26]. Some studies used the co-opetition game model. Luo et al. (2016) focused on two competing manufactures under cap-and-trade, investigated the optimal pricing and green technology investment in purely competition and co-opetition respectively[27]. Niu et al. (2019) developed a co-opetition supply chain consisting of an original equipment manufacturer and a competitive contract manufacture, analyzed incentive alignment of the economic and environmental sustainability in a co-opetition model[28].

The following literature studies the decision-making of construction industry enterprises under cap-and-trade. Jiang et al. (2018) focused on a two echelon supply chain consisting of a supplier and a prefabricated building manufacturer, constructed a stackelberg model and obtained the optimal pricing and carbon emission decisions[29]. Jiang et al. (2019) focused on the two-level construction supply chain composed of the GC and the SC and constructed the profit distribution model of the construction supply chain under cap-and-trade policy and obtained the optimal profit distribution ratio and the level of carbon emission reduction efforts for both parties[30], this study considered three cases: the pure competition, the co-opetition and the pure cooperation, compared the optimal decisions in three cases. On the basis of this literature, our study pay attention to the fairness concern behavior, investigate the impact of fairness concern on the decision-making in construction supply chain.

### Operational decisions considering fairness concern

Fehr (1999) argued that people are willing to pay a high price to punish free riders in an agreement because people have fairness concern[11]. Ho (2014) studied two cases of fairness concern: when the retailer has fairness concern about the supplier and when the retailer has fairness concern about another retailer. The optimal wholesale price was studied for both cases[31]. Zhou et al. (2016) considered a low-carbon supply chain channel with one manufacturer and one retailer and designed advertising and carbon emission reduction cost sharing contracts based on fairness concerns[32]. Jiang et al. (2017) discussed the initial allocation of carbon emission permits in various Chinese provinces and established the initial inter-provincial carbon emission permit allocation model take into account fairness concerns[33]. Zhang and Wang (2018) studied the impact of fairness concern on the coordination of a three-party supply chain and constructed a game model for a duopoly supply chain. The results showed that the horizontal fairness of enterprises will have a direct impact on their own wholesale prices and competitors’ prices and profits[34]. Chang and Hu (2018) constructed an incentive contract model considering risk capital and analyzed the impact of fairness concern on the contract design[35]. Li et al. (2018) focused on two-echelon supply chain with a fair-neutral manufacturer and a fairness-concerned retailer and explored carbon emission reduction decisions and prices[36].

The research on fairness concern mentioned above has not been introduced into a construction supply chain. Studies of construction supply chain enterprises decision considering fairness concern are as follows: Kadefors et al. (1999) studied the impact of fairness factors on engineering projects. The research suggested that the owner should be as reasonable as possible when designing contracts to avoid contractors feeling that their contract is unfair[37]. Meng et al. (2018) considered the fairness concern of a construction project’s participants. The study built an agent-based model on revenue sharing negotiation and further analyzed the impact of fairness concern on the success rate of the negotiations[38]. An et al. (2018) investigated a construction supply chain composed of owners and designers, established a profit distribution negotiation model considering fairness concern and analyzed the influence of fairness concern on the optimal profit distribution[39]. Jiang and Yuan (2019) constructed a profit distribution model that considers fairness concern and determined the optimal decisions for the supply chain members[40]. The study incorporated fairness concern in the F-S model and only considered an SC with fairness concern. On this basis, we improve the method for incorporating fairness concern and adopt Nash bargaining model to describe the reference point of the fairness concern. Further, we study the two parties’ optimal decisions in three situations: only the SC has fairness concern, only the GC has fairness concern, and both parties have fairness concern.

On the other hand, most of the existing studies on fairness concern adopt the F-S model, but this method does not consider the ability, contribution, and cognitive ability of both parties[41]. Nash pioneered a new game method called the Nash Bargaining Model[42]. Bruyn (2008) analyzed the impact of fairness concern on the bargaining behavior among supply chain entities and found that the results of bargaining under fairness concern change significantly[43]. Du et al. (2014) based on the Nash bargaining game model, incorporated fairness concerns, and examined a dyadic supply chain in which both the supplier and the retailer. The study assumed both sides have fairness concern and investigated a newsvendor problem of the supply chain[41]. Li (2018) considered Nash bargaining power and fairness concerns, and studies pricing and quality decisions[44].

### Incentive mechanism of the construction supply chain

To achieve goal optimization, a bonus incentive is one of the commonly used methods in construction supply chain[45–47]. Berends et al. (2000) proposed a type of cost-plus-incentive fee contract to share the cost risk and conducted eight case studies to prove its feasibility[48]. Bubshait et al. (2003) indicated that the owner can induce the initiative to control the factors affecting the construction cost, duration and productivity of the project by signing an incentive contract with the contractor[49]. As the main agents in the project, the GC and SC play a very important role in the success of the project. Shr and Chen (2004) established a construction determine incentive model for the highway construction project and obtained the optimal incentive amount for the owner to pay the contractor[50]. Fan et al. (2018) designed an incentive plan for green buildings and analyzed the impact of transaction costs on green building performance[51]. Chan et al. (2008) studied the Hong Kong subway construction project and found that the contract with the total price processing period bonus can significantly improve the project performance[52]. Jiang et al. (2010) designed a reasonable bonus incentive contract for the optimization of highway project duration and further determined the optimal benchmark for a construction period reward[53]. Meng and Gallagher (2012) studied the impact of bonus incentive contracts on construction goals in UK and Irish construction projects. The combination of incentives and penalties can be used not only for cost targets, but also for time, quality and environmental targets[54]. Hosseinian and Carmichael (2013) established an bonus incentive model for the owner and contractor based on different risk preferences and extended it to multi-target (time, cost and safety) bonus incentive models in non-cooperative situations[55]. Kerkhove and Vanhoucke (2017) developed a multi-objective of cost, duration, safety and built a decision-making system that included incentive contract design, multi-objective trade-offs and performance assessment[56]. Shi et al. (2018) based on a mega prefabricated construction and built an incentive model with reputational concerns[57].

The studies above lay a good foundation for the construction of carbon emission reduction incentive contract model. However, most of the existing research on the incentive mechanism of the construction supply chain bonus focuses on the application of bonus incentives to the optimization of the duration and quality and on the lack of reducing emissions.

## Model descriptions and assumptions

This study investigates carbon emission reduction and a profit distribution mechanism for a construction supply chain consisting of a GC and SC. The owner and the GC sign a fixed total price plus bonus contract, and the GC also signs a fixed total price plus bonus contract with the SC. First, the GC and SC will determine the profit distribution ratio during the contract negotiation process. Then, according to this ratio, the two parties will determine their own carbon emission reduction efforts with the goal of maximizing their profits. The study takes into account cap-and-trade policy. The government regulates the carbon emissions cap per unit of area of the structure. If this cap is exceeded, the firm needs to purchase carbon credits in the carbon trading market. If there is surplus, the firm can sell the remaining carbon emission rights[58].

In practice, the negotiation power of the GC is usually stronger than that of the SC. Therefore, the traditional profit distribution model is often dominated by the GC, the GC decides the profit distribution ratio with the goal of maximizing profit. The SC only has the right to accept or waive the agreement. This model will decrease the SC’s motivation. This problem has been notices in the existing research and a co-opetition model has been proposed, which proves that the performance of both parties will be better than in the pure competition model[30]. The co-opetition model allows the two parties to determine the profit distribution ratio with the goal of maximizing the profit of the supply chain and then to determine their own efforts according to the goal of maximizing their own profits. This is in line with the actual contract negotiation procedure between the parties. Therefore, this study also uses the co-opetition model. To describe our model, we use the notations presented in Table 1.

**Table 1:** Notations of parameters and variables.

Decision variables	Descriptions
***λ***	Distribution ratio of emission reduction bonuses
***e*_*i*_**	Unit carbon emission reduction of the GC/SC respectively, *i* = 1,2.
**Parameters**	
***P*_*i*_**	Fixed price portion of enterprise *i*, *i* = 1,2. The values 1 and 2 represent the GC and the SC, respectively.
***μ***	GC bonus coefficient obtained by the owner.
***s***	Construction area.
***c*_1*i*_**	Traditional construction cost for GC/SC before the carbon emission reduction input, *i* = 1,2.
**c_1_**	Total cost of construction supply chain before the carbon emission reduction input, *c*_1_ = *c*_11_ + *c*_12_.
***e*_0_**	Initial unit carbon emissions of GC/SC.
***e*_*s*_**	Carbon emission cap.
***E*_0_**	Carbon trading volume in the carbon trading market.
***t*_*i*_**	Carbon emission reduction cost coefficient of the GC/SC, *i* = 1,2.
***k***	Unit carbon emission price in the carbon trading market.
***γ*_*i*_**	Fairness concern coefficient of GC/ SC, *i* = 1,2.
**A**	*k* + *μ*
***B***	*k*(*e*_0_ − *e*_*s*_)
***C***	2 + *γ*_1_ + *γ*_2_
***u*_*i*_**	2 + *γ*_*i*_, *i* = 1,2
***v*_12_**	$\frac{A}{2u_{1}t_{2}}$
***v*_21_**	$\frac{A}{2u_{2}t_{1}}$
***M***	$\frac{2+\gamma_{1}u_{2}-\gamma_{2}^{2}}{C}$
***N***	$\frac{2+\gamma_{2}u_{1}-{\gamma_{1}}^{2}}{C}$

In addition, the assumptions in this study are as follows.

1. GC/SC’s carbon emission reduction cost is $t_{i}e_{i}^{2}$, *i* = 1,2. This assumption means that the GC/SC’s carbon emission reduction cost is a quadratic function of *e*_*i*_. This setting is popular in the literature[59,60].
2. To represent the actual situation in the construction industry and to facilitate calculation, the government’s carbon emission allowance is calculated based on the carbon emissions per unit area. We assumed that the government-defined carbon emission cap is *e*_*s*_. The initial carbon emission per unit construction area is *e*_0_. The carbon emission trading quantity of the unit construction area that needs to be traded is *E*_0_ = *e*_0_ − *e*_1_ − *e*_2_ − *e*_*s*_. Carbon trading gains/costs for the supply chain after production are given by *skE*_0_. When *E*_0_ > 0, this indicates that the contractor’s carbon emission reduction cannot meet the cap and it is necessary to purchase carbon emission rights in the external trading market. When *E*_0_ < 0, this means that the contractor’s carbon emission reduction not only meets the cap, but also has a remainder which can be sold.
3. *P*_1_ > *P*_2_, the GC’s fixed price portion is higher than that of the SC. This is consistent with what is true in practice.
4. In the variable representation above, subscripts 1, 2 represent the GC and SC respectively. *sc* represents the construction supply chain. The first letter of the superscript indicates whether the GC has fairness concern and the second letter indicates whether the SC has fairness concern, where *f* stands for fairness concern, *n* stands for fair-neutral. For example, *nf* stands for the GC is fair-neutral and the SC is fairness concern.

## Base model

In this section, the GC and SC do not consider fairness concern, which means that both parties are fair-neutral. This section is the base model of this study.

The GC’s profit function is:

$$\Pi_{1}(\lambda ,e_{1})=P_{1}-P_{2}+\lambda [\mu s(e_{1}+e_{2})-skE_{0}]-st_{1}e_{1}^{2}{-c}_{11}$$ (1)

The SC’s profit function is:

$$\Pi_{2}(\lambda ,e_{2})=P_{2}+(1-\lambda )[\mu s(e_{1}+e_{2})-skE_{0}]-st_{2}e_{2}^{2}{-c}_{12}$$ (2)

The construction supply chain’s profit function is:

$$\Pi_{sc}(e_{1},e_{2})={\Pi_{1}(\lambda ,e_{1})+\Pi_{2}(\lambda ,e_{2})=P}_{1}+\mu s(e_{1}+e_{2})-skE_{0}-st_{1}e_{1}^{2}-st_{2}e_{2}^{2}-c_{1}$$ (3)

The GC’s decision problem is to decide the optimal carbon emission reduction and maximize his profit *Π*_1_(*λ*, *e*_1_). Therefore, the GC’s decision problem is:

$$\max\;\Pi_{1}^{nn}(\lambda ,e_{1})$$ (4)

$$s.t.\;{e_{0}-e_{1}-e_{2}=E}_{0}+e_{s}$$ (5)

Similarly, the SC’s decision problem is:

$$\text{max}\;\Pi_{2}^{nn}(\lambda ,e_{2})$$ (6)

**Proposition 1**. In this section, the GC’s carbon emissions reduction under cap-and-trade is:

$$e_{1}^{nn}(\lambda )=\frac{\lambda }{2t_{1}}A$$ (7)

The SC’s carbon emissions reduction under cap-and-trade is:

$$e_{2}^{nn}(\lambda )=\frac{1-\lambda }{2t_{2}}A$$ (8)

**Proof**. From Eqs (1) and (2), $\frac{\partial \Pi_{1}^{nn}(\lambda ,e_{1})}{\partial e_{1}}=-2st_{1}e_{1}+A\lambda s$, $\frac{\partial \Pi_{2}^{nn}(\lambda ,e_{2})}{\partial e_{2}}=-2st_{2}e_{2}+As(\lambda -1)$ can be got and $\frac{\partial^{2}\Pi_{1}^{nn}(\lambda ,e_{1})}{\partial e_{1}^{2}}=-2st_{1}<0$, $\frac{\partial^{2}\Pi_{2}^{nn}(\lambda ,e_{2})}{\partial e_{2}^{2}}=-2st_{2}<0$. Let $\frac{\partial \Pi_{1}^{nn}(\lambda ,e_{1})}{\partial e_{1}}=0$, $\frac{\partial \Pi_{2}^{nn}(\lambda ,e_{2})}{\partial e_{2}}=0$. $e_{1}^{nn}(\lambda )=\frac{\lambda }{2t_{1}}A$, $e_{2}^{nn}(\lambda )=\frac{1-\lambda }{2t_{2}}A$ can be obtained. $\frac{\partial e_{1}^{nn}(\lambda )}{\partial k}=\frac{\lambda }{2t_{1}}>0$, $\frac{\partial e_{2}^{nn}(\lambda )}{\partial k}=\frac{1-\lambda }{2t_{2}}>0$. This completes the proof.

Proposition 1 shows that the unit carbon emissions reduction of the GC and the SC is closely related to the distribution ratio (*λ*). The GC’s carbon emission reduction is proportional to *λ* and the SC’s carbon emission reduction is inversely proportional to *λ*.

**Proposition 2**. In this section, the optimal profit distribution ratio under cap-and-trade is $\lambda^{nn}=\frac{t_{2}}{t_{1}+t_{2}}$.

**Proof**. Substitute Eqs (7) and (8) into (1). Then we can get $\frac{\partial \Pi_{sc}^{nn}(\lambda )}{\partial \lambda }=\frac{\mu^{2}s}{2t_{1}}-\frac{\mu^{2}s}{2t_{2}}-\frac{2\mu s(\lambda \mu +k)}{4t_{1}}+\frac{2\mu s[(1-\lambda )\mu +k]}{4t_{2}}+\frac{\mu ks}{2t_{1}}-\frac{\mu ks}{2t_{2}}$, $\frac{\partial^{2}\Pi_{sc}^{nn}(\lambda )}{{\partial \lambda }^{2}}=-\frac{2\mu^{2}s}{4t_{1}}-\frac{2\mu^{2}s}{4t_{2}}<0$, that is, *Π*_*sc*_(*λ*) is concave in *λ*. Let $\frac{\partial \Pi_{sc}^{nn}(\lambda )}{\partial \lambda }=0$, then $\lambda^{nn}=\frac{t_{2}}{t_{1}+t_{2}}$. This completes the proof.

We use co-opetition model to describe the profit distribution model of both parties. Proposition 2 shows that *λ*^*nn*^ is only related to *t*_*i*_. The higher *t*_2_, that is, the lower the efficiency of SC’s research and development, the lower the profit from SC. Therefore, if SC wants to achieve higher profits, it must improve R&D efficiency.

## Decision model with fairness concern

In this section, we formulate the models with that three different types of fairness concerns: the SC has fairness concern (*nf*), the GC has fairness concern (*fn*) and both sides have fairness concern (*ff*).

### Decision model when the SC has fairness concern

This section considers the case where the SC has fairness concern and the GC is fair-neutral, that is *γ*_1_ = 0 and *γ*_2_ > 0. This study improves the traditional fairness reference framework by constructing the Nash bargaining model. This is a new perspective for representing fairness concern in a construction supply chain. The utility of firms with fairness concern depends on the benefits realized and the fairness reference point. For simplicity and practicality, a linear form is used for the utility of each member in the construction supply chain as follows. Because the GC is fair-neutral, so the utility function of the GC is:

$$U_{1}^{nf}(\lambda ,e_{1})=\Pi_{1}$$ (9)

The utility function of the SC is:

$$U_{2}^{nf}(\lambda ,e_{2})=\Pi_{2}+\gamma_{2}(\Pi_{2}-{\overset{-}{\Pi }}_{2})=(1+\gamma_{2})\Pi_{2}-\gamma_{2}{\overset{-}{\Pi }}_{2}$$ (10)

The utility function of the construction supply chain is:

$$U_{sc}^{nf}(e_{1},e_{2})=U_{1}^{nf}(\lambda ,e_{1})+U_{2}^{nf}(\lambda ,e_{2})$$ (11)

According to the definition of a Nash bargaining game, the Nash solution is the following model.

$$U_{p}^{nf}=U_{1}^{nf}U_{2}^{nf}=\Pi_{1}[(1+\gamma_{2})\Pi_{2}-\gamma_{2}{\overset{-}{\Pi }}_{2}]$$ (12)

$$maxU_{p}^{nf}$$ (13)

$$s.t.\;\Pi_{1}+\Pi_{2}=\Pi_{sc}$$ (14)

$$U_{1}^{nf},U_{2}^{nf}>0$$ (15)

The SC’s fairness reference solution is:

$${\overset{-}{\Pi }}_{2}^{nf}=\frac{1+\gamma_{2}}{2+\gamma_{2}}\Pi_{sc}$$ (16)

Therefore, the SC’s utility function is:

$$U_{2}^{nf}(\lambda ,e_{2})=(1+\gamma_{2})\Pi_{2}-\frac{\gamma_{2}(1+\gamma_{2})}{2+\gamma_{2}}\Pi_{sc}=\frac{2+2\gamma_{2}}{u_{2}}\Pi_{2}-\frac{\gamma_{2}+\gamma_{2}^{2}}{u_{2}}\Pi_{1}$$ (17)

The utility function of the construction supply chain is:

$$U_{sc}^{nf}(e_{1},e_{2})=\frac{2+2\gamma_{2}}{u_{2}}\Pi_{2}+\frac{2-\gamma_{2}^{2}}{u_{2}}\Pi_{1}$$ (18)

The GC’s decision problem is:

$$\max\;U_{1}^{nf}(\lambda ,e_{1})$$ (19)

$$s.t.\;{e_{0}-e_{1}-e_{2}=E}_{0}+e_{s}$$ (20)

The SC’s decision problem is:

$$\text{max}\;U_{2}^{nf}(\lambda ,e_{2})$$ (21)

**Proposition 3**. When the SC has fairness concern, the GC’s carbon emissions reduction is:

$$e_{1}^{nf}(\lambda )=\frac{\lambda }{2t_{1}}A$$ (22)

The SC’s carbon emissions reduction is:

$$e_{2}^{nf}(\lambda )=\frac{\left[2(1-\lambda )-\lambda \gamma_{2}\right]}{4t_{2}}A$$ (23)

**Proof**. From Eq (6), $\frac{\partial U_{1}^{nf}(\lambda ,e_{1})}{\partial e_{1}}=s[-2e_{1}t_{1}+A\lambda ]$. Let $\frac{\partial U_{1}^{nf}(\lambda ,e_{1})}{\partial e_{1}}=0$, we can get $e_{1}^{nf}(\lambda )=\frac{A\lambda }{2t_{1}}$. From Eq (7), $\frac{\partial U_{2}^{nf}(\lambda ,e_{2})}{\partial e_{2}}=\frac{-s(1+\gamma_{2})[4e_{2}t_{2}+{A\gamma }_{2}\lambda +2A(\lambda -1)]}{u_{2}}$. Let $\frac{\partial U_{2}^{nf}(\lambda ,e_{2})}{\partial e_{2}}=0$, we can get $e_{2}^{nf}(\lambda )=\frac{\left[2(1-\lambda )-\lambda \gamma_{2}\right]}{4t_{2}}A$. $\frac{\partial e_{1}^{nf}(\lambda )}{\partial \lambda }=\frac{A}{2t_{1}}>0$, $\frac{\partial e_{2}^{nf}(\lambda )}{\partial \lambda }=\frac{-A(2+\gamma_{2})}{4t_{2}}<0$, $\frac{\partial e_{2}^{nf}(\lambda )}{\partial \gamma_{2}}=\frac{-A\lambda }{4t_{2}}<0$. This completes the proof.

From this proposition, it can be concluded that the GC’s carbon emission reduction increases with the increase of the distribution ratio (*λ*). The SC’s carbon emission reduction decrease as the distribution ratio increases, it also decreases as the fairness concern coefficient (*γ*_2_) increases.

**Proposition 4**. When the SC is fairness concern, the optimal profit distribution ratio is $\lambda^{nf}=\frac{2A^{2}[2t_{2}(1+\gamma_{2})-u_{2}t_{1}\gamma_{2}]+4Bu_{2}t_{1}t_{2}\gamma_{2}}{A^{2}[-t_{1}(\gamma_{2}^{3}+3\gamma_{2}^{2}-4]+2t_{2}{(\gamma_{2}^{2}+4\gamma }_{2}+2)}$.

**Proof**. Substitute $e_{1}^{nf}(\lambda )$ and $e_{2}^{nf}(\lambda )$ into (2) and (3) respectively, we can get $\Pi_{1}^{nf}(\lambda ,e_{1}^{nf})$ and $\Pi_{2}^{nf}(\lambda ,e_{2}^{nf})$. Then substitute $\Pi_{1}^{nf}(\lambda ,e_{1}^{nf})$ and $\Pi_{2}^{nf}(\lambda ,e_{2}^{nf})$ into (8). Let $\frac{\partial U_{sc}^{nf}(e_{1},e_{2})}{\partial \lambda }=0$, then we can get the result. This completes the proof.

From Proposition 4, we can obtain the optimal profit distribution ratio of the two parties in this case. We can see that the distribution ratio is closely related to the SC’s fairness concern coefficient (*γ*_2_) and to the carbon emission reduction cost coefficient (*t*_*i*_).

### Decision model when the GC has fairness concern

Most traditional studies are based on the Stackelberg game, and the GC usually is usually the leader. The leader side tends to be in a favorable position and will not make decisions that are detrimental to himself, so he does not have fairness concern. However, this study is based on the co-opetition model, which studies the situation where the position difference between the two parties is small and both parties have the right to speak. Furthermore, this study represents the fairness concern using the Nash bargaining theory. The fairness reference point is the Nash bargaining equilibrium point, not the profit of the other parties. Both parties will perceive unfairness if a difference exists between their own utility and the Nash reference point, which is irrelevant with both parties’ status. Therefore, this section considers that the GC has fairness concern, that is *γ*_1_ > 0 and *γ*_2_ = 0.

The utility function of the GC is:

$$U_{1}^{fn}(\lambda ,e_{1})=\Pi_{1}+\gamma_{1}(\Pi_{1}-{\overset{-}{\Pi }}_{1})=(1+\gamma_{1})\Pi_{1}-\gamma_{1}{\overset{-}{\Pi }}_{1}$$ (24)

The utility function of the SC is:

$$U_{2}^{fn}(\lambda ,e_{2})=\Pi_{2}$$ (25)

The utility function of the construction supply chain is:

$$U_{sc}^{fn}(e_{1},e_{2})=U_{1}^{fn}(\lambda ,e_{1})+U_{2}^{fn}(\lambda ,e_{2})$$ (26)

According to the definition of a Nash bargaining game, the Nash solution is the solution of the following model.

$$U_{p}^{fn}=U_{1}^{fn}(\lambda ,e_{1})U_{2}^{fn}(\lambda ,e_{2})=\Pi_{2}[(1+\gamma_{1})\Pi_{1}-\gamma_{1}{\overset{-}{\Pi }}_{1}]$$ (27)

$$max\;U_{p}^{fn}$$ (28)

$$s.t.\;\Pi_{1}+\Pi_{2}=\Pi_{sc}$$ (29)

$$U_{1}^{fn},U_{2}^{fn}>0$$ (30)

The GC’s fair reference solution is:

$${\overset{-}{\Pi }}_{1}^{fn}=\frac{1+\gamma_{1}}{2+\gamma_{1}}\Pi_{sc}$$ (31)

In this case, the GC’s utility function is:

$$U_{1}^{fn}(\lambda ,e_{1})=(1+\gamma_{1})\Pi_{1}-\frac{\gamma_{1}(1+\gamma_{1})}{2+\gamma_{1}}\Pi_{sc}=\frac{2+2\gamma_{1}}{u_{1}}\Pi_{1}-\frac{\gamma_{1}+\gamma_{1}^{2}}{u_{1}}\Pi_{2}$$ (32)

The utility function of the construction supply chain is:

$$U_{sc}^{fn}(e_{1},e_{2})=\frac{2+2\gamma_{1}}{u_{1}}\Pi_{1}+\frac{2-\gamma_{1}^{2}}{u_{1}}\Pi_{2}$$ (33)

The GC’s decision problem is:

$$\max\;U_{1}^{fn}(\lambda ,e_{1})$$ (34)

$$s.t.\;{e_{0}-e_{1}-e_{2}=E}_{0}+e_{s}$$ (35)

The SC’s decision problem is:

$$\max\;U_{2}^{fn}(\lambda ,e_{2})$$ (36)

**Proposition 5**. When the GC has fairness concern, the GC’s carbon emissions reduction is:

$$e_{1}^{fn}(\lambda )=\frac{u_{1}\lambda -\gamma_{1}}{4t_{1}}A$$ (37)

The SC’s carbon emissions reduction is:

$$e_{2}^{fn}(\lambda )=\frac{1-\lambda }{2t_{2}}A$$ (38)

**Proof**: From Eqs (8) and (9), Let $\frac{\partial U_{1}(\lambda ,e_{1})}{\partial e_{1}}=0$, $\frac{\partial U_{1}(\lambda ,e_{1})}{\partial e_{1}}=\frac{s(1+\gamma_{1})(-4e_{1}t_{1}+A\gamma_{1}(\lambda -1)+2A\lambda )}{2+\gamma_{1}}=0$. So $e_{1}^{fn}(\lambda )=\frac{{A(u}_{1}\lambda -\gamma_{1})}{4t_{1}}$. Let $\frac{\partial U_{2}(\lambda ,e_{2})}{\partial e_{2}}=0$, $\frac{\partial U_{2}(\lambda ,e_{2})}{\partial e_{2}}=-s[2Ae_{2}t_{2}(\lambda -1)]=0$, so $e_{2}^{fn}(\lambda )=\frac{1-\lambda }{2t_{2}}A$. We can get $\frac{\partial e_{1}^{fn}(\lambda )}{\partial \lambda }=\frac{2+\gamma_{1}}{4t_{1}}A>0$, $\frac{\partial e_{1}^{fn}(\lambda )}{\partial \gamma_{1}}=\frac{\lambda -1}{4t_{1}}A<0$, $\frac{\partial e_{2}^{fn}(\lambda )}{\partial \lambda }=\frac{-A}{2t_{1}}<0$. This completes the proof.

The GC’s carbon emission reduction is directly proportional to the distribution ratio (*λ*) and the SC’s carbon emission reduction is inversely proportional to *λ*. As the level of the GC’s fairness concern increases, his carbon emission reductions decrease.

**Proposition 6**: When the GC has fairness concern, the optimal profit distribution ratio is $\lambda^{fn}=\frac{A^{2}[2{u_{1}t}_{1}\gamma_{1}+u_{1}t_{2}(-\gamma_{1}^{2}+\gamma_{1}+2)]-4Bu_{1}t_{1}t_{2}\gamma_{1}}{A^{2}[2t_{1}(\gamma_{1}^{2}+4\gamma_{1}+2)+u_{1}^{2}t_{2}(1-\gamma_{1})]}$.

**Proof**: Substitute (37) and (38) into (1) and (2) respectively, we can get $\Pi_{1}^{fn}(\lambda ,e_{1}^{nf})$ and $\Pi_{2}^{fn}(\lambda ,e_{2}^{nf})$. Then substitute $\Pi_{1}^{fn}(\lambda ,e_{1}^{nf})$ and $\Pi_{2}^{fn}(\lambda ,e_{2}^{nf})$ into (11). Let $\frac{\partial U_{sc}^{fn}(e_{1},e_{2})}{\partial \lambda }=0$, then we can get the result. This completes the proof.

From Proposition 6, we can obtain the optimal profit distribution ratio of the two parties in this case. We can see that the distribution ratio is closely related to the GC’s fairness concern coefficient (*γ*_1_) and the carbon emission reduction cost coefficient (*t*_*i*_).

### Decision model when both sides have fairness concern

The section will further study the profit distribution of the construction supply chain based on the assumption that both the GC and SC have fairness concern.

The utility function of the GC is:

$$U_{1}^{ff}(\lambda ,e_{1})=\Pi_{1}+\gamma_{1}(\Pi_{1}-{\overset{-}{\Pi }}_{1})=(1+\gamma_{1})\Pi_{1}-\gamma_{1}{\overset{-}{\Pi }}_{1}$$ (39)

The utility function of the SC is:

$$U_{2}^{ff}(\lambda ,e_{2})=\Pi_{2}+\gamma_{2}(\Pi_{2}-{\overset{-}{\Pi }}_{2})=(1+\gamma_{2})\Pi_{2}-\gamma_{2}{\overset{-}{\Pi }}_{2}$$ (40)

The utility function of the construction supply chain is:

$$U_{SC}(e_{1},e_{2})=U_{1}(\lambda ,e_{1})+U_{2}(\lambda ,e_{2})$$ (41)

*γ*_1_ and *γ*_2_ represent the fairness concern coefficient of the GC and SC respectively, reflecting the degree of emphasis on fairness of each by party, with *γ*_1_ ≥ 0 and *γ*_2_ ≥ 0. ${\overset{-}{\Pi }}_{1}$ and ${\overset{-}{\Pi }}_{2}$ are the fairness reference point, clearly *Π*_1_ + *Π*_2_ = *Π*_*sc*_ and ${\overset{-}{\Pi }}_{1}+{\overset{-}{\Pi }}_{2}=\Pi_{sc}$.

According to the definition of Nash bargaining game, the Nash solution is the solution of the following model:

$$U_{p}^{ff}=U_{1}^{ff}U_{2}^{ff}=[(1+\gamma_{1})\Pi_{1}-\gamma_{1}{\overset{-}{\Pi }}_{1}][\left(1+\gamma_{2})(\Pi_{sc}-\Pi_{1})-\gamma_{2}(\Pi_{sc}-{\overset{-}{\Pi }}_{1}\right)]$$ (42)

$$maxU_{p}^{ff}$$ (43)

$$s.t.\;\Pi_{1}+\Pi_{2}=\Pi_{sc}$$ (44)

$$U_{1}^{ff},U_{2}^{ff}>0$$ (45)

We can obtain the benchmark for the GC as [41, 42]:

$${\overset{-}{\Pi }}_{1}^{ff}=\frac{1+\gamma_{1}}{2+\gamma_{1}+\gamma_{2}}\Pi_{sc}=\frac{1+\gamma_{1}}{C}\Pi_{sc}$$ (46)

The benchmark for the SC is

$${\overset{-}{\Pi }}_{2}^{ff}=\frac{1+\gamma_{2}}{2+\gamma_{1}+\gamma_{2}}\Pi_{sc}=\frac{1+\gamma_{2}}{C}\Pi_{sc}$$ (47)

In this case, the utility function of the GC is:

$$U_{1}^{ff}(\lambda ,e_{1})=(1+\gamma_{1})\Pi_{1}-\gamma_{1}(\frac{1+\gamma_{1}}{2+\gamma_{1}+\gamma_{2}}\Pi_{sc})=\frac{\left(1+\gamma_{1})({u_{2}\Pi }_{1}-\gamma_{1}\Pi_{2}\right)}{C}$$ (48)

The utility function of the SC is:

$$U_{2}^{ff}(\lambda ,e_{2})=(1+\gamma_{2})\Pi_{2}-\gamma_{2}(\frac{1+\gamma_{2}}{2+\gamma_{1}+\gamma_{2}}\Pi_{sc})=\frac{\left(1+\gamma_{2})(u_{1}\Pi_{2}-\gamma_{2}\Pi_{1}\right)}{C}$$ (49)

The utility function of the construction supply chain is:

$$U_{sc}^{ff}=U_{1}^{ff}(\lambda ,e_{1})+U_{2}^{ff}(\lambda ,e_{2})=M\Pi_{1}+N\Pi_{2}$$ (50)

The GC’s decision problem is:

$$\max\;U_{1}^{ff}(\lambda ,e_{1})$$ (51)

$$s.t.\;{e_{0}-e_{1}-e_{2}=E}_{0}+e_{s}$$ (52)

The SC’s decision problem is:

$$\max\;U_{2}^{ff}(\lambda ,e_{2})$$ (53)

**Proposition 7**: When both sides have fairness concern, the GC’s carbon emissions reduction is:

$$e_{1}^{ff}=v_{21}(C\lambda -\gamma_{1})$$ (54)

The SC’s carbon emissions reduction is:

$$e_{2}^{ff}=v_{12}[u_{1}(1-\lambda )-{\lambda \gamma }_{2}]$$ (55)

**Proof**: From Eqs (10) and (11), we can get $\frac{\partial U_{1}^{ff}(\lambda ,e_{1})}{\partial e_{1}}=\frac{-s(1+\gamma_{1})\{u_{2}[2t_{1}e_{1}-A\lambda ]+A\gamma_{1}(1-\lambda )\}}{C}$ and $\frac{\partial U_{2}^{ff}(\lambda ,e_{2})}{\partial e_{2}}=\frac{-s(1+\gamma_{2})\{u_{1}[2t_{2}e_{2}-A(1-\lambda )]+A\gamma_{2}\lambda \}}{C}$. Let $\frac{\partial U_{1}^{ff}(\lambda ,e_{1})}{\partial e_{1}}=0$ and $\frac{\partial U_{2}^{ff}(\lambda ,e_{2})}{\partial e_{2}}=0$, we can obtain $e_{1}^{ff}=v_{21}(C\lambda -\gamma_{1})$, $e_{2}^{ff}=v_{12}[u_{1}(1-\lambda )-{\lambda \gamma }_{2}]$. $\frac{\partial e_{1}^{ff}}{\partial \lambda }=Cv_{21}>0$, $\frac{\partial e_{2}^{ff}}{\partial \lambda }=-v_{12}(u_{1}+\gamma_{2})<0$. We can get the result.

From Proposition 7, we can obtain the optimal carbon emission reductions for both parties in this situation. It can be seen that the GC’s carbon emission reduction is proportional to *λ* and that of the SC is inversely proportional to *λ*.

**Proposition 8**: When both sides have fairness concern, the optimal profit distribution ratio is $\lambda^{ff}=\frac{2Ct_{1}(Nu_{1}v_{12}^{2}-Mv_{21}^{2}\gamma_{1})+[A({v_{21}\gamma_{1}-u}_{1}v_{12})+B](N-M)+ACN(v_{21}-v_{12})}{2C^{2}(Mt_{1}v_{21}^{2}+Nt_{2}v_{12}^{2})+2AC(N-M)(v_{21}-v_{12})}$

**Proof**: The solution process is similar to Proposition 6. Substitute $e_{1}^{ff}(\lambda )$ and $e_{2}^{ff}(\lambda )$ into (1) and (2) respectively, we can get $\Pi_{1}^{ff}(\lambda ,e_{1}^{ff})$ and $\Pi_{2}^{ff}(\lambda ,e_{2}^{ff})$. Then substitute $\Pi_{1}^{ff}(\lambda ,e_{1}^{ff})$ and $\Pi_{2}^{ff}(\lambda ,e_{2}^{ff})$ into (14). Let $\frac{\partial U_{sc}^{ff}(e_{1},e_{2})}{\partial \lambda }=0$, then we can get the result.

From Proposition 8, we can obtain the optimal profit distribution ratio of the two parties in this case. We can see that the distribution ratio is closely related to the fairness concern coefficient of the GC/SC (*γ*_*i*_) and the carbon emission reduction cost coefficient (*t*_*i*_).

## Numerical analysis

A numerical analysis of the construction supply chain is presented here to illustrate how the fairness concern affects the GC’s and SC’s decisions. The support data for this section is saved in S1 File.

In this section, numerical analysis is provided to examine the impact of the fairness concern on firms’ operation decisions. More specifically, we discuss the impact of the GC’s and SCs’ fairness concern coefficients (*γ*_1_ and *γ*_2_, respectively) on the maximum profits of the GC, the SC, and the supply chain. We set *e*_0_ = 9, *e*_*s*_ = 7, *t*_1_ = 6, *t*_2_ = 8, *k* = 30, *μ* = 5, *P*_1_ = 3200000, *P*_2_ = 1200000, *s* = 1000, *C*_11_ = 1500000, *C*_12_ = 1000000.

### Impact of SC’s fairness concern on optimal decisions and profits

To observe the change in the optimal decisions and profits as the degree of the SC’s fairness concern increases, we set *γ*_2_ ∈ [0,2].

**Fig 1:**
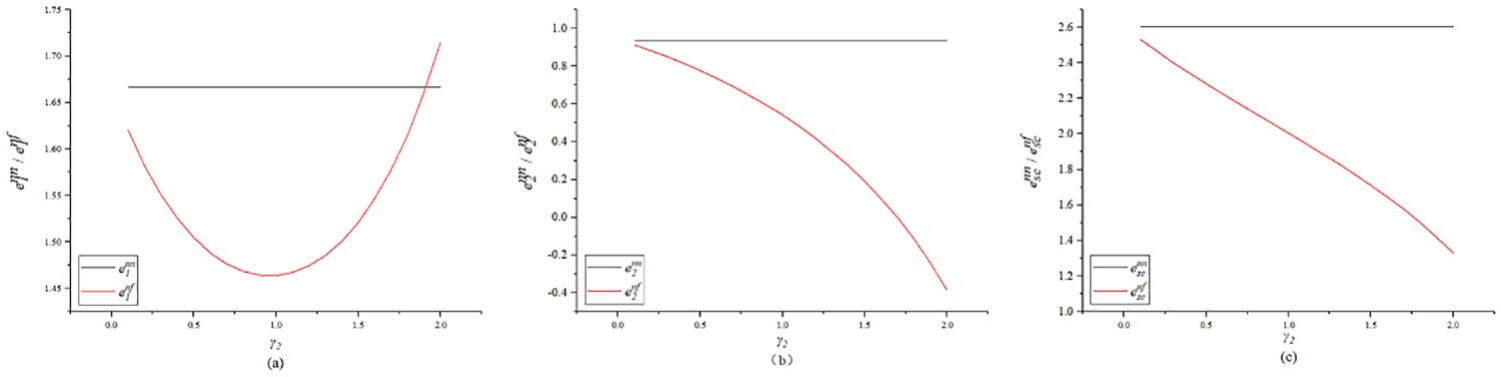
Impact of *γ*_2_ on the unit carbon emission reduction.

Fig 1 indicates the impact of *γ*_2_ on the unit carbon emission reduction. Fig 1(a) presents the trend in the GC’s carbon emission reduction. The figure shows that as *γ*_2_ increases, the GC’s carbon emission reduction first decrease and then increases. Fig 1(b) presents the trend in the SC’s carbon emission reduction. The figure shows that, as *γ*_2_ increases, the SC’s carbon emission reduction decreases. Because the game model used in this study is the co-opetition model, the two sides first make a decision by maximizing their own utility and then allocate a proportion to the overall profit of the supply chain. When the SC has fairness concern, his own utility will decrease, so the emission reduction will also decrease, and GC’s emission reduction is positively correlated with λ. When the SC has fairness concern, the GC’s bonus shares will decrease, so the GC’s emission reduction will first decrease until it reaches the lowest point in the figure, it is the minimum amount that the GC can tolerate. When *γ*_2_ increases further, the GC has to pay an additional reduction cost to avoid high transaction costs in the external market. It can be seen that in the co-opetition mode, the GC needs to find an SC with a low or zero fairness concern. Fig 1(c) is a trend graph of the total carbon emission reductions of the construction supply chain. It shows that, when the SC has fairness concern, the total carbon emission reductions of the supply chain decrease. In summary, the SC’s fairness concern will affect the performance of the supply chain’s carbon emission reduction, which will hinder the expansion of carbon emission reduction in the construction industry.

**Fig 2:**
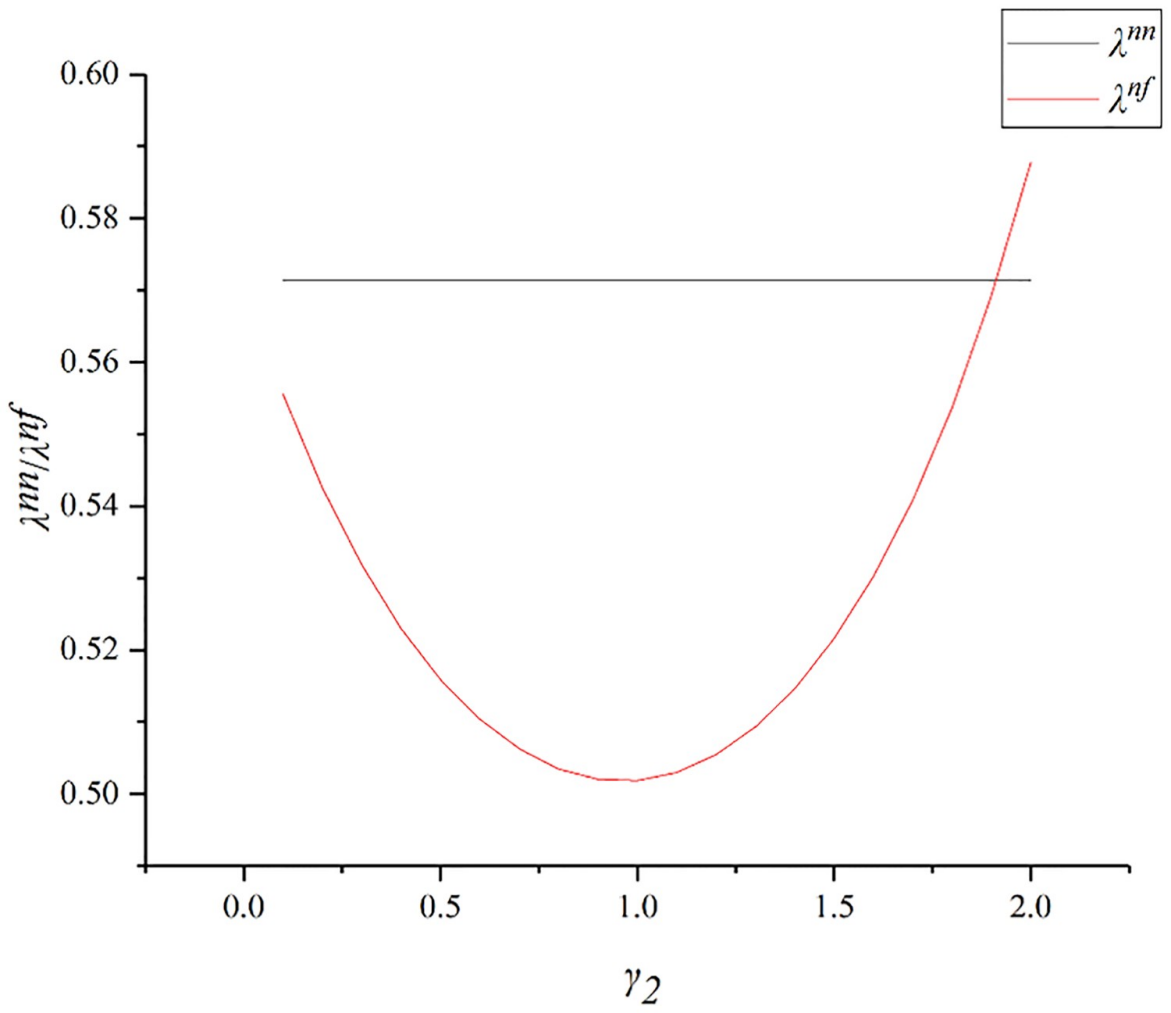
Impact of *γ*_2_ on *λ*.

Fig 2 shows the impact of *γ*_2_ on the distribution ratio (*λ*). The GC’s bonus ratio is *λ* and that of the SC is 1 − *λ*. Therefore, it can be concluded from the figure that SC with fairness concern will receive a larger share of the bonus. However, when the degree of fairness is too large, it can be seen in the figure that *γ*_2_ > 1, and the SC’s shares obtained by the SC will begin to decrease. This is because the GC can initially tolerate the SC’s fairness concern and will meet the SC’s requirements. However, when the SC’s degree of fairness is too high, the GC will only pursue his own interests.

**Fig 3:**
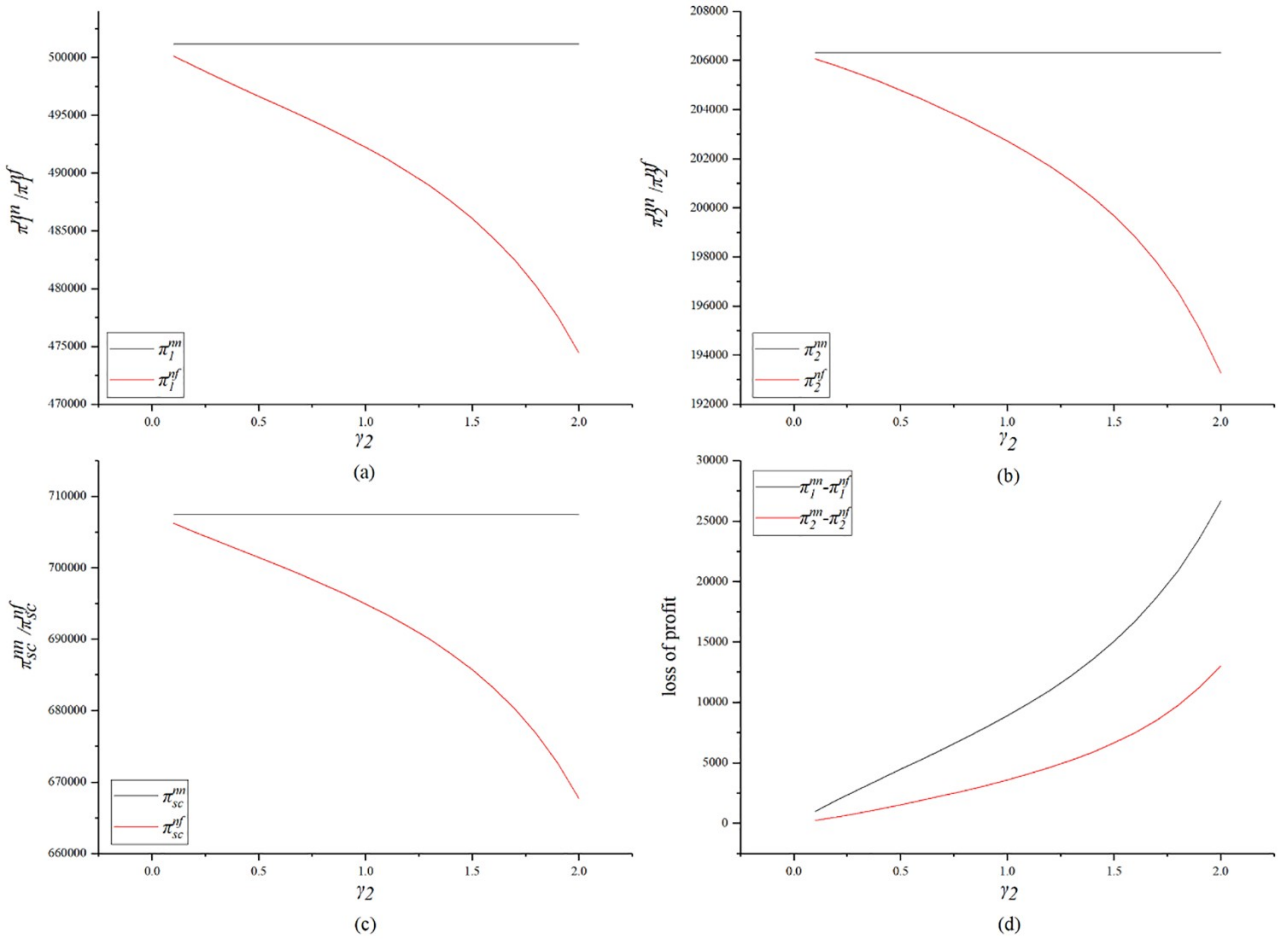
Impact of *γ*_2_ on the profits.

Fig 3 shows the impact of *γ*_2_ on profits. Fig 3(a), 3(b) and 3(c) indicate that the SC’s fairness concern will damage both sides’ profits and will also damage the overall profit of the supply chain. This trend is due to the fact that in the model description of this study, the owner’s carbon emission reduction incentives are linearly related to the supply chain’s carbon emission reductions. The carbon emission reduction incentives given by the GC to the SC are also linearly related to the SC’s carbon emission reduction. Therefore, when both the supply chain’s carbon emission reductions are decrease in magnitude, both parties’ carbon emission reduction bonuses are reduced, which ultimately leads to lower profits. Fig 3(d) is a loss trend graph of the profit of each party. It can be seen from the figure that the GC’s profit loss is higher than that of the SC with considering the fairness concern of the SC. It can be seen that the SC’s fairness concern will harm the interests of both parties. In summary, the SC’s fairness concern can damage the profit of the supply chain. Even if the SC can obtain a higher profit share for a certain degree of fairness, both parties’ profits will still be damaged.

### Impact of GC’s fairness concern on optimal decisions and profits

To observe the change in profit as the degree of the GC’s fairness concern increases, we set *γ*_1_ ∈ [0,2].

**Fig 4:**
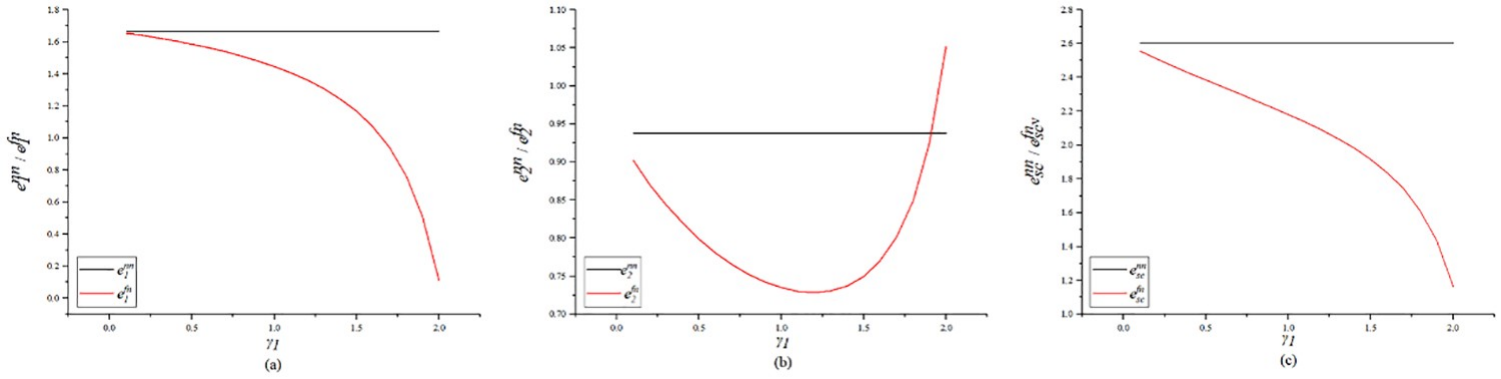
Impact of *γ*_1_ on the unit carbon emission reduction.

Fig 4 shows the impact of *γ*_1_ on carbon emission reductions. Fig 4(a) shows that when *γ*_1_ increases, the GC’s carbon emission reductions will decrease. Fig 4(b) shows that, as *γ*_1_ increases, the SC’s carbon emission reductions first decline and then increase. Fig 4(c) shows that when *γ*_1_ increases, the total carbon emission reductions of the supply chain decrease. In summary, the fairness of the GC will also affect the carbon emission reduction performance of the construction supply chain.

**Fig 5:**
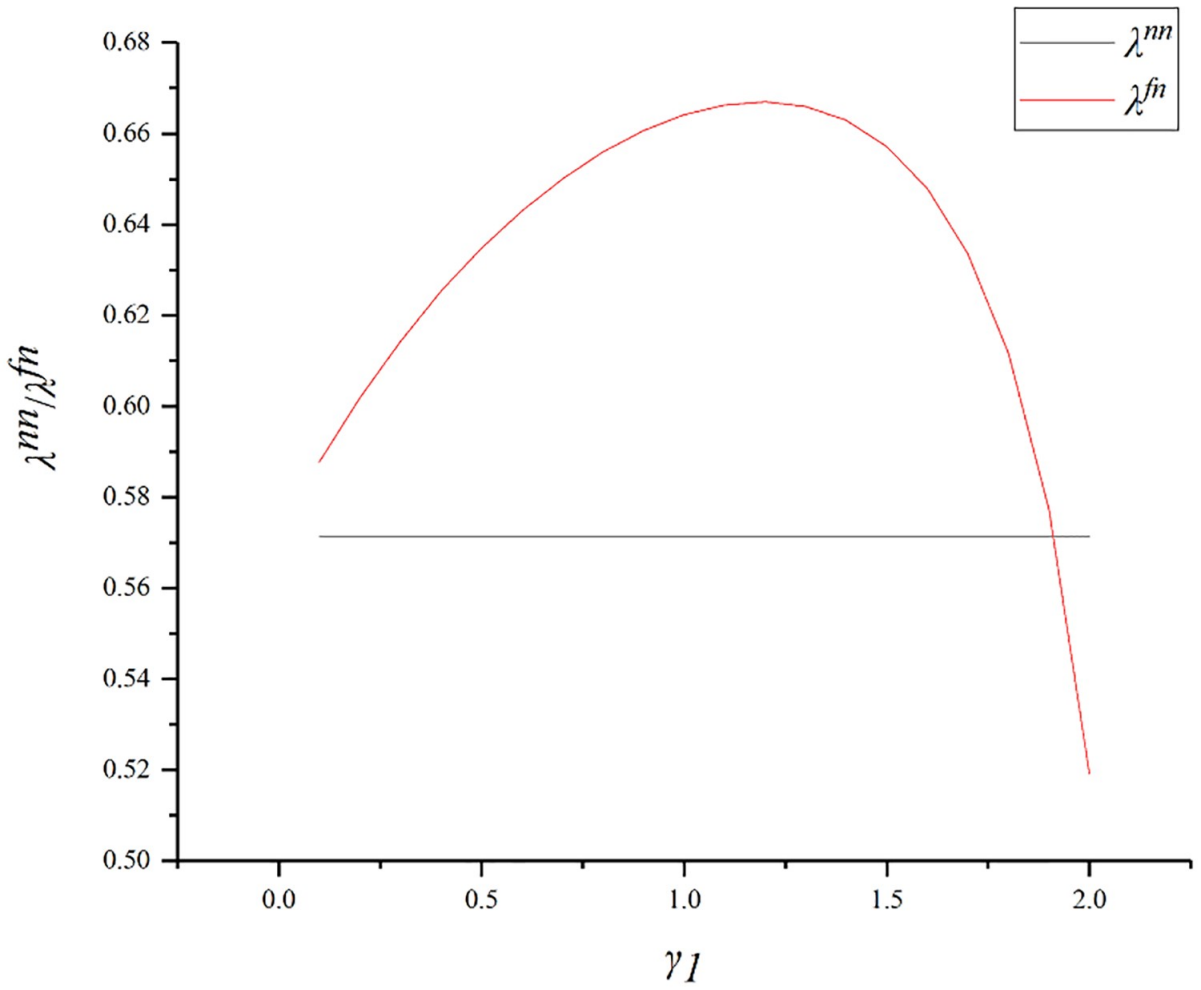
Impact of *γ*_1_ on *λ*.

Fig 5 shows the impact of *γ*_1_ on the distribution ratio (*λ*). As *γ*_1_ increases, *λ*^*fn*^ first rises and then falls. When *γ*_1_ is close to 2, the GC’s profit share will be less than what it would be without fairness concerns. As can be seen from the figure, the GC’s share is always higher than the SC’s, and rises up to 0.67. This shows that, although the GC has certain advantages, these will not be too large. This is because the ratio of bonuses allocated to the two parties aims to maximize the profit of the supply chain, and there is no case where the SC’s share is extremely small when the GC pays too much attention to the fairness concern. Therefore, as an SC is often the weaker party, using co-opetition to negotiate can maximize his own profits.

**Fig 6:**
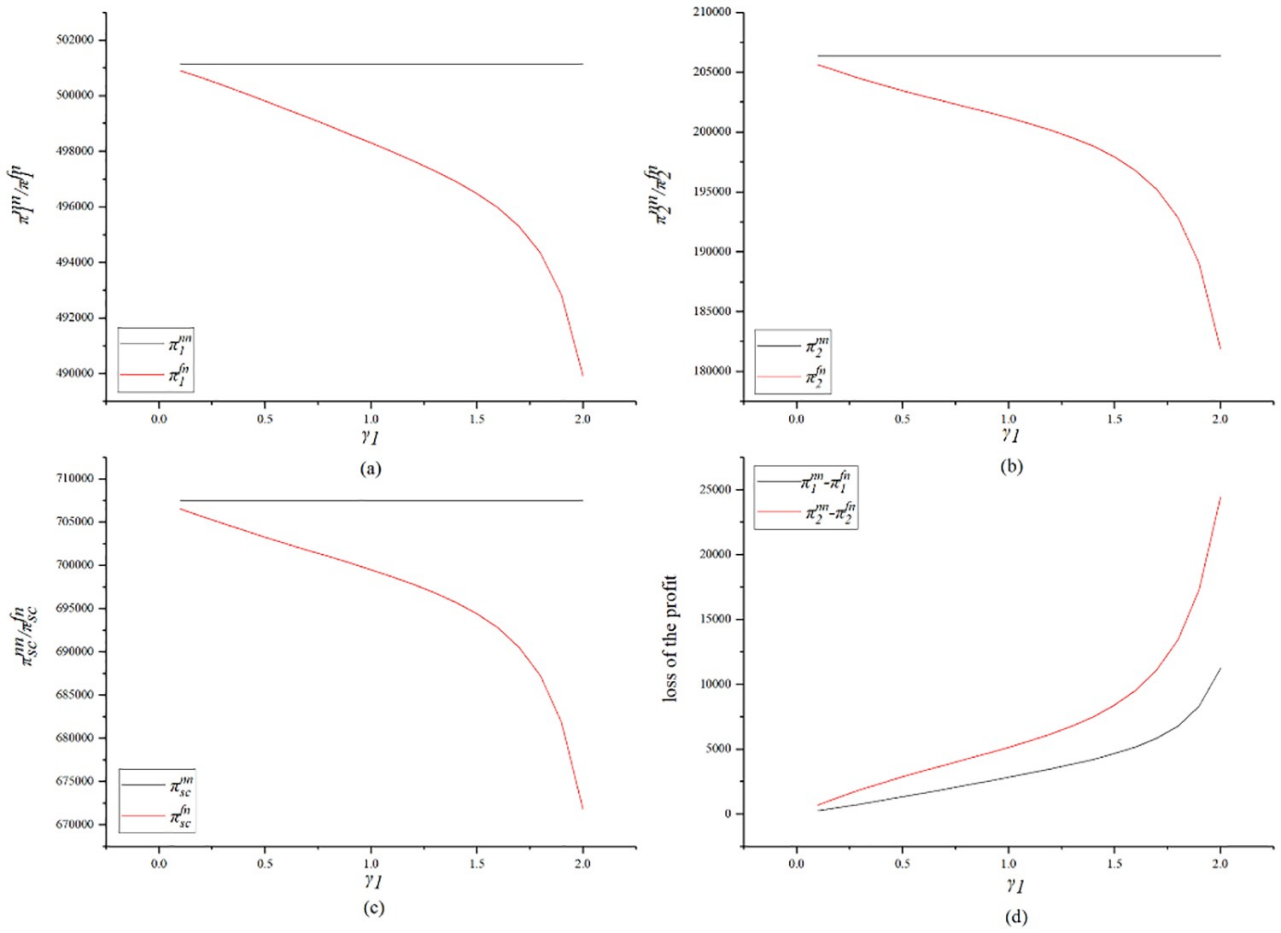
Impact of *γ*_1_ on the profits.

Fig 6 shows the impact of *γ*_1_ on profits. Fig 6(a), 6(b) and 6(c) indicate that *γ*_1_ results in damages for both parties and the supply chain. Fig 6(c) shows that the SC’s losses are greater than the GC’s if the GC has fairness concern. It is worth noting that when *γ*_1_ rises from 0 to 2, the supply chain profit falls from 706534.9 to 671804 and the profit loss is 37430.9. When *γ*_2_ rises from 0 to 2, the supply chain profit drops from 706214.3 to 667750.6 and the profit loss is 38463.7. It can be seen that the SC’s fairness concern has a greater impact on the supply chain’s profit.

### Impact of both sides’ fairness concern on optimal decisions and profits

To observe the change in profit when the degree of both sides’ fairness concern is increasing simultaneously, we set *γ*_1_ ∈ [0,2], *γ*_2_ ∈ [0,2].

**Fig 7:**
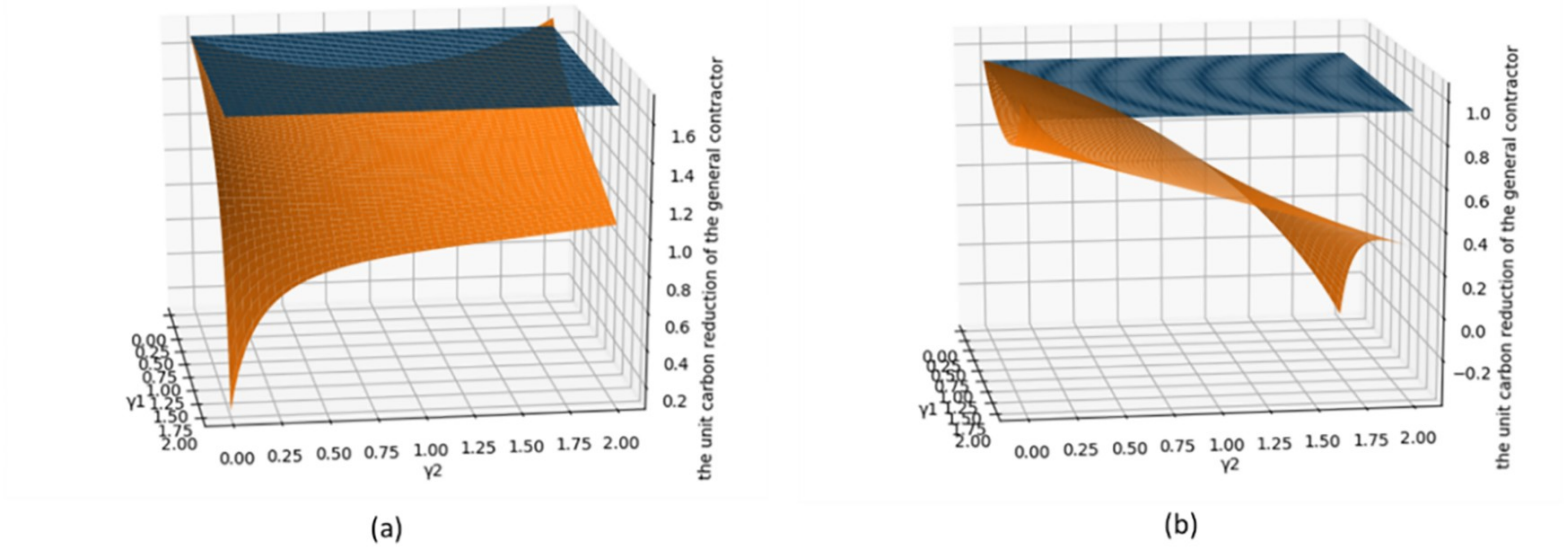
Impact of the both sides’ fairness concern on the unit carbon emission reduction.

Fig 7 shows a comparison between the carbon reduction when no fairness concerns exist, and when fairness concerns exist in both parties. Fig 7(a) shows the impact of both parties’ fairness concern on the GC’s carbon emission reductions. As the level of the SC’s fairness concern increases, the GC’s carbon emission reduction first decreases and then slightly increases. As the level of the GC’s fairness concern increases, his carbon emission reductions decrease. Fig 7(a) shows the impact of both parties’ fairness concern on the GC’s carbon emission reductions. Fig 7(b) shows that the impact of both parties’ fairness concern on the SC’s carbon emission reductions. The fairness concern will reduce both parties’ carbon emission reductions. As the level of SCs’ fairness concern increases, the SC’s carbon emission reduction decrease, and are even lower than those without a low-carbon input. As the level of *γ*_1_ increases, the SC’s emission reduction will first decrease and then increase. This is different from the conclusion in the literature (Zhang et al.(2019)[61]), namely that the follower’s fairness concern do not affect the carbon emissions of the product. This is because that study examined a situation in which the status of the two sides is very different, and the Stackelberg game is used. Further, the market demand in that study was related to low carbon. We adopt the co-opetition game, which is applicable to the situation where the status of the two parties is not very different. The two parties aim to maximizing the profit of the supply chain, and both parties have the right to negotiate. In this case, the fairness concern in either party will affect the emission reductions and profits of both parties.

**Fig 8:**
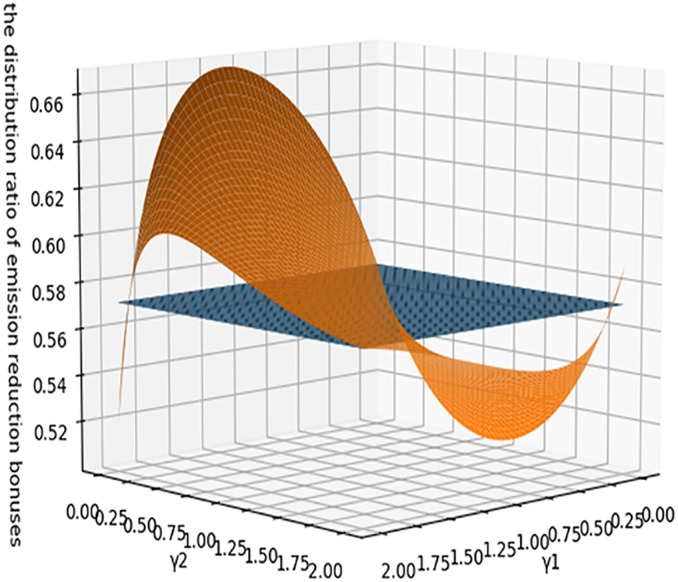
Impact of the both sides’ fairness concern on λ.

Fig 8 presents the trend in λ when considering the degree of fairness concern of both parties simultaneously. From the figure we can see that, when the two parties have the same degree of fairness concern, the distribution ratio is the same as in the base model. When a party has a high level of fairness, it will obtain a higher share, in the range [0.5, 0.68], and there will be no extreme values. This shows that, in a co-opetition game, there is fairness concern, but distribution ratio is relatively reasonable, and there will be no situation where one party has full advantage over the other.

**Fig 9:**
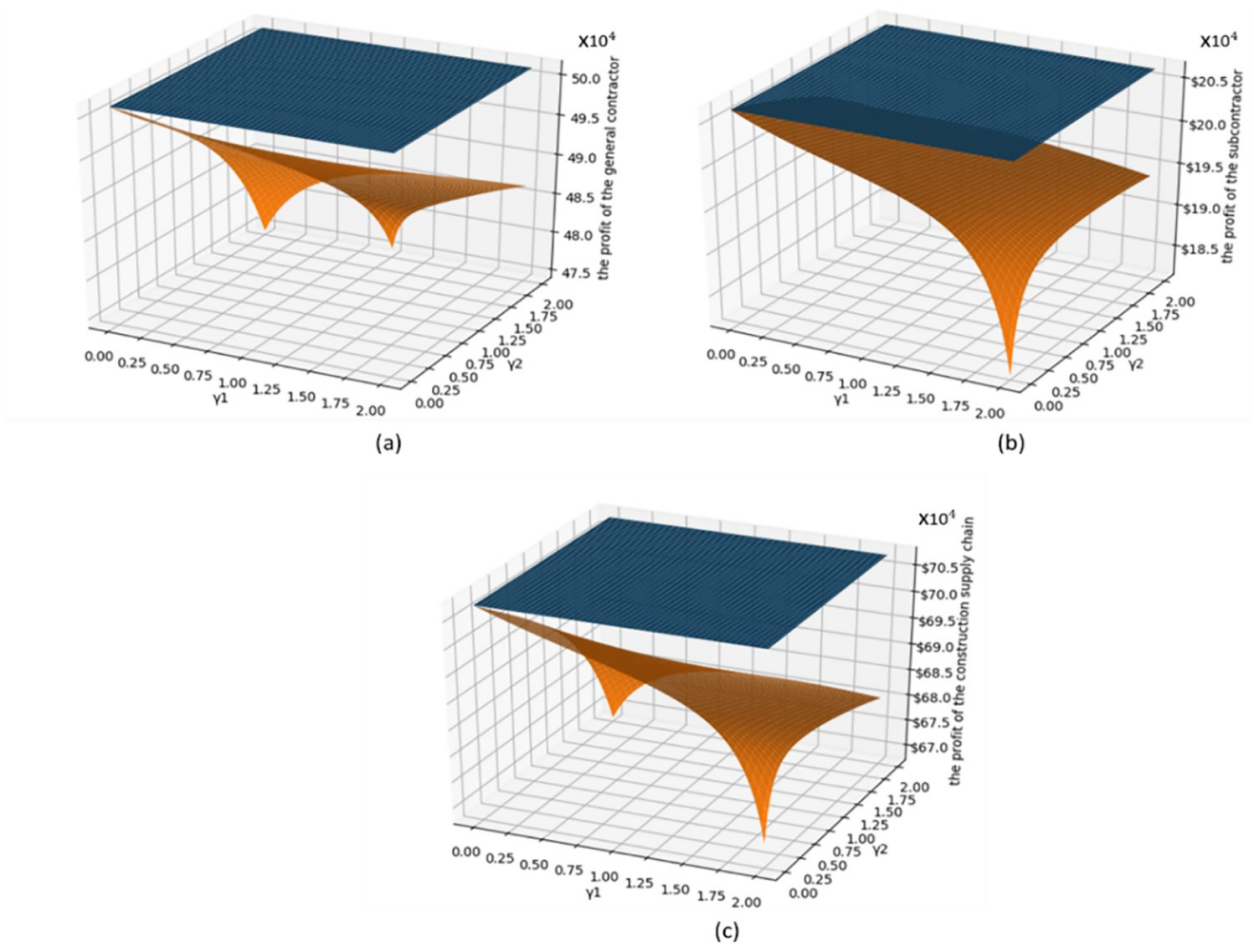
Impact of the both sides’ fairness concern on profit.

Fig 9 shows a comparison of the profits when no fairness concern exists, and when fairness concerns exists in both parties. From Fig 9(a) and 9(b), as the level of fairness of the two parties increases, their profits decrease. From Fig 9(c), as the level of fairness of the two parties increase, the overall profit of the construction supply chain also decreases. This shows that fairness concern can hurt the profits of both parties.

## Conclusions and future research

China is vigorously promoting cap-and-trade policy and accelerating the improvement of the carbon emission trading market. As construction is one of the highly polluting industries, it is necessary to study the decisions in a construction supply chain under cap-and-trade. Fairness concern behavior has also begun to be of interest. In this study, a two-echelon construction supply chain comprising a GC and an SC is considered. The utility functions considering the fairness concern in the construction supply chain are formulate by using the Nash bargaining solution. Then, the profit distribution model for both parties is constructed in three cases: only the SC has fairness concern, only the GC has fairness concern, and both parties have fairness concern. The optimal carbon emission reduction decisions and the optimal profit distribution ratio of the two parties in the three cases are obtained. The impact of fairness concern on carbon emission reductions and profit is also examined.

### Managerial implications

Our study has three main managerial implications. First, when the GC and SC are fair-neutral, carbon emission reductions are closely related to the R&D cost efficiency (*t*_*i*_) of the two parties and the owner’s bonus factor. In order to obtain higher bonuses, the GC and SC should improve their carbon emission reduction efficiency; if the owner pursues a “greener” building, the coefficient of the bonus should moderately increase. Second, it is concluded that fairness concern behavior can seriously affect supply chain performance. If the owner offers carbon emission reduction bonuses, this will encourage the GC and SC to increase their carbon emission reduction efforts, but when supply chain firms have fairness concern, because of the gap between the actual profits they receive and their reference points, the motivation for reducing emissions will ultimately decrease. The profits of the individual firms and the overall supply chain are both damaged. Third, based on the numerical analysis, we can conclude that the SC’s fairness concern has a greater negative impact on supply chain performance. Therefore, when selecting the SC, the GC should try to choose a fair-neutral SC, or take some measures to reduce the SC’s level of fairness. This study is based on the co-opetition game. We find that the co-opetition game is beneficial to the weaker side.

### Limitations and future research

This study considers the impact of fairness concern on the carbon emission reduction and profit distribution of the construction supply chain under cap-and-trade policy and enriches the literature on the construction supply chain. It would be interesting to extend our study to examine the influence of fairness concern in the construction supply chain consisting of a GC and multiple SCs. Further, other members of the construction supply chain can also be included, such as the owner and the designer. On the other hand, with the increasing awareness regarding social environmental protection, it is also possible to study the impact of consumers’ low-carbon demand on the decision-making of construction supply chain enterprises. These will greatly enrich the current study and make our research more realistic. Moreover, this study only investigates the impact of fairness concern on the decision-making of supply chain firms and does not give a quantitative method to mitigate the adverse effects; developing such a method will be the future research direction of the author.

## Supporting information

S1 File Data file. (XLSX)

## References

[1] Liu B, Holmbom M, Segerstedt A, Chen W. Effects of carbon emission regulations on remanufacturing decisions with limited information of demand distribution. International Journal of Production Research. 2015; 53(2):532–548.

[2] Morrissey J, Horne RE. Life cycle cost implications of energy efficiency measures in new residential buildings. Energy and Buildings. 2011; 43(4):915–924.

[3] International Energy Agency releases global carbon dioxide emissions report(2018) [Internet]. http://www.tanjiaoyi.com/article-26529-1.html

[4] How much has China’s carbon emissions increased in 2018? [Internet]. http://www.tanjiaoyi.com/article-26181-1.html

[5] Briscoe G, Dainty A. Construction supply chain integration: an elusive goal? Supply Chain Management: An International Journal, 2005, 10(4):319–326.

[6] Vrijhoef R, Koskela L. The four roles of supply chain management in construction. European Journal of Purchasing & Supply Management. 2000; 6(3):169–178.

[7] Ofori G. Greening the construction supply chain in Singapore. European Journal of Purchasing & Supply Management. 2000; 6(3):195–206.

[8] Jiang W, Yuan L, Wu LJ, Zhou Y. Pricing and carbon emission strategy of supply chain with cap and trade. IOP Conference Series: Materials Science and Engineering. IOP Publishing, 2018; 439(3):032046.

[9] Xu X, He P, Xu H, Zhang Q. Supply chain coordination with green technology under cap-and-trade regulation. International Journal of Production Economics. 2017; 183:433–442.

[10] Leung DYC, Yung D, Ng A, Leung MKH, Chan A. An overview of emissions trading and its prospects in Hong Kong. Environmental Science & Policy. 2009; 12(1):92–101.

[11] Fehr E, Schmidt KM. A theory of fairness, competition, and cooperation. The Quarterly Journal of Economics. 1999; 114(3):817–868.

[12] Liu W, Wang S, Zhu D, Wang D, Shen X. Order allocation of logistics service supply chain with fairness concern and demand updating: model analysis and empirical examination. Annals of Operations Research. 2018; 268(1):177–213.

[13] Du S, Wei L, Zhu Y, Nie T. Peer-regarding fairness in supply chain. International Journal of Production Research. 2018; 56(10):3384–3396.

[14] Pu X, Gong L, Han G. A feasible incentive contract between a manufacturer and his fairness-sensitive retailer engaged in strategic marketing efforts. Journal of Intelligent Manufacturing. 2019; 30(1):193–206.

[15] Rabin M. Incorporating Fairness into Game Theory and Economics. The American Economic Review. 1993; 83(5):1281–1302.

[16] Scheer LK, Kumar N, Steenkamp J-BEM. Reactions to perceived inequity in U.S. and dutch interorganizational relationships. Academy of Management Journal. 2003; 46(3):303–316.

[17] Forsythe R, Horowitz JL, Savin NE, Sefton M. Fairness in Simple Bargaining Experiments. Games and Economic Behavior. 1994; 6(3):347–369.

[18] Nie T, Du S. Dual-fairness supply chain with quantity discount contracts. European Journal of Operational Research. 2017; 258(2):491–500.

[19] Wang Y, Chen W, Liu B. Manufacturing/remanufacturing decisions for a capital-constrained manufacturer considering carbon emission cap and trade. Journal of Cleaner Production. 2017; 140:1118–1128.

[20] Yang L, Ji J, Wang M, Wang Z. The manufacturer’s joint decisions of channel selections and carbon emission reductions under the cap-and-trade regulation. Journal of Cleaner Production. 2018; 193:506–523.

[21] Hugo A, Pistikopoulos EN. Environmentally conscious long-range planning and design of supply chain networks. Journal of Cleaner Production. 2005; 13(15):1471–1491.

[22] The Carbon Trust. Carbon footprints in the supply chain: the next step for business [Internet]. 2006. https://www.mendeley.com/catalogue/carbon-footprints-supply-chain-next-step-business/

[23] Du SF, Zhu LL, Liang L, Ma F. Emission-dependent supply chain and environment-policy-making in the ‘cap-and-trade’ system. Energy Policy, 2013, 57: 61–67.

[24] Xu X, He P, Xu H, Zhang Q. Supply chain coordination with green technology under cap-and-trade regulation. International Journal of Production Economics. 2017; 183:433–442.

[25] Xia L, Guo T, Qin J, Yue X, Zhu N. Carbon emission reduction and pricing policies of a supply chain considering reciprocal preferences in cap-and-trade system. Annals of Operations Research. 2018; 268(1):149–175.

[26] Wang X, Xue M, Xing L. Analysis of carbon emission reduction in a dual-channel supply chain with cap-and-trade regulation and low-carbon preference. Sustainability. 2018; 10(3):580.

[27] Luo Z, Chen X, Wang X. The role of co-opetition in low carbon manufacturing. European Journal of Operational Research, 2016, 253(2): 392–403.

[28] Niu B, Mu Z, Chen L, et al Coordinate the economic and environmental sustainability via procurement outsourcing in a co-opetitive supply chain[J]. Resources, Conservation and Recycling, 2019, 146: 17–27.

[29] Jiang W, Wu L, Zhou Y. Pricing and Carbon Reduction Mode for Prefabricated Building Supply Chain with Cap and Trade International Conference on Management Science and Engineering Management. Springer, Cham, 2018: 1417–1427.

[30] Jiang W, Lu WF, Xu QW. Profit distribution model for construction supply chain with cap-and-trade policy. Sustainability. 2019; 11(4):1215.

[31] Ho TH, Su X, Wu Y. Distributional and peer-induced fairness in supply chain contract design. Production and Operations Management. 2014; 23(2):161–175.

[32] Zhou Y, Bao M, Chen X, Xu X. Co-op advertising and emission reduction cost sharing contracts and coordination in low-carbon supply chain based on fairness concerns. Journal of Cleaner Production. 2016; 133:402–413.

[33] Jiang H, Shao X, Zhang X, Bao J. A study of the allocation of carbon emission permits among the provinces of china based on fairness and efficiency. Sustainability. 2017; 9(11):2122.

[34] Zhang T, Wang X. The impact of fairness concern on the three-party supply chain coordination. Industrial Marketing Management. 2018; 73:99–115.

[35] Chang J, Hu Z. Venture capital contracting with double-sided moral hazard and fairness concerns [Internet]. Mathematical Problems in Engineering. 2018. https://www.hindawi.com/journals/mpe/2018/5296350/abs/

[36] Li Q, Xiao T, Qiu Y. Price and carbon emission reduction decisions and revenue-sharing contract considering fairness concerns. Journal of Cleaner Production. 2018; 190:303–314.

[37] Kadefors A. Client-contractor relations: How fairness considerations and interests influence contractor variation negotiations. Proceedings IGLC-7, 1999:231–240.

[38] Meng Q, Chen J, Qian K. The complexity and simulation of revenue sharing negotiation based on construction stakeholders [Internet]. Complexity.2018 https://www.hindawi.com/journals/complexity/2018/5698170/abs/

[39] Xiaowei A, Huimin L, Ojuri O, Zhuofu W, Jiyong D. Negotiation model of design optimization profit distribution with fairness concerns in construction projects. KSCE Journal of Civil Engineering. 2018; 22(7):2178–2187.

[40] Jiang W, Yuan L. Profit distribution model of green building supply chain with fairness preferences and cap-and-trade policy. IOP Conference Series: Earth and Environmental Science. 2019; 237: 052043.

[41] Du S, Nie T, Chu C, Yu Y. Newsvendor model for a dyadic supply chain with Nash bargaining fairness concerns. International Journal of Production Research. 2014; 52(17):5070–5085.

[42] Nash JF Jr. The Bargaining Problem. Econometrica. 1950; 18:155–162.

[43] De Bruyn A, Bolton G E. Estimating the influence of fairness on bargaining behavior. Management science. 2008; 54(10):1774–1791.

[44] Li J, Lu J, Wang Q, Li C. Quality and pricing decisions in a two-echelon supply chain with Nash bargaining fairness concerns. discrete dynamics in nature and society [Internet]. 2018; https://www.hindawi.com/journals/ddns/2018/4267305/abs/

[45] Shi Q, Zhu J, Li Q. Cooperative evolutionary game and applications in construction supplier tendency [Internet]. Complexity. 2018. https://www.hindawi.com/journals/complexity/2018/8401813/abs/

[46] Zeng W, Wang H, Li H, Zhou H, Wu P, Le Y. Incentive mechanisms for supplier development in mega construction projects. IEEE Transactions on Engineering Management. 2019; 66(2):252–265.

[47] Amirreza M, Mehdi MM. Financial-based incentive plan to reduce construction waste. Journal of Construction Engineering and Management. 2018; 144(5):04018029.

[48] Berends TC. Cost plus incentive fee contracting—experiences and structuring. International Journal of Project Management. 2000; 18(3):165–171.

[49] Bubshait AA. Incentive/disincentive contracts and its effects on industrial projects. International Journal of Project Management. 2003; 21(1):63–70.

[50] Shr JF, Chen WT. Setting maximum incentive for incentive/disincentive contracts for highway projects. Journal of Construction Engineering and Management. 2004; 130(1):84–93.

[51] Fan K, Chan EHW, Qian QK. Transaction costs (TCs) in green building (GB) incentive schemes: Gross Floor Area (GFA) Concession Scheme in Hong Kong. Energy Policy. 2018; 119:563–573.

[52] Chan APC, Chan WM, Fan CN, Lam PTI, Yeung JFY. Achieving partnering success through an incentive agreement: lessons learned from an underground railway extension project in Hong Kong. Journal of Management in Engineering. 2008; 24(3):128–137.

[53] Yi J, Chen HX, Li S. Determination of contract time and incentive and disincentive values of highway construction projects. International Journal of Construction Education and Research. 2010; 6(4):285–302.

[54] Meng X, Gallagher B. The impact of incentive mechanisms on project performance. International Journal of Project Management. 2012; 30(3):352–362.

[55] Hosseinian SM, Carmichael DG. Optimal incentive contract with risk-neutral contractor. Journal of Construction Engineering and Management. 2013; 139(8):899–909.

[56] Kerkhove LP, Vanhoucke M. A parallel multi-objective scatter search for optimising incentive contract design in projects. European Journal of Operational Research. 2017; 261(3):1066–1084.

[57] Shi Q, Zhu J, Hertogh M, Sheng Z. Incentive mechanism of prefabrication in mega projects with reputational concerns. Sustainability. 2018; 10(4):1260.

[58] Groenenberg H, Blok K. Benchmark-based emission allocation in a cap-and-trade system. Climate Policy. 2002; 2(1):105–109.

[59] Chen X, Wang X, Chan HK. Manufacturer and retailer coordination for environmental and economic competitiveness: A power perspective. Transportation Research Part E: Logistics and Transportation Review. 2017; 97:268–281.

[60] Chen X, Luo Z, Wang X. Impact of efficiency, investment, and competition on low carbon manufacturing. Journal of Cleaner Production. 2017; 143:388–400.

[61] Zhang L, Zhou H, Liu Y, Lu R. Optimal environmental quality and price with consumer environmental awareness and retailer’s fairness concerns in supply chain. Journal of Cleaner Production. 2019; 213:1063–1079.

